# Aldosterone: a mediator of retinal ganglion cell death and the potential role in the pathogenesis in normal-tension glaucoma

**DOI:** 10.1038/cddis.2013.240

**Published:** 2013-07-04

**Authors:** E Nitta, K Hirooka, K Tenkumo, T Fujita, A Nishiyama, T Nakamura, T Itano, F Shiraga

**Affiliations:** 1 Department of Ophthalmology, Kagawa University Faculty of Medicine, 1750-1 Ikenobe, Miki, Kagawa, Japan; 2 Department of Pharmacology, Kagawa University Faculty of Medicine, 1750-1 Ikenobe, Miki, Kagawa, Japan; 3 Department of Neurobiology, Kagawa University Faculty of Medicine, 1750-1 Ikenobe, Miki, Kagawa, Japan; 4 Department of Ophthalmology, Okayama University Graduate School of Medicine, Density and Pharmaceutical Sciences, 2-5-1 Shikata-cho, Okayama, Japan

**Keywords:** aldosterone, retinal ganglion cell, normal-tension glaucoma, intraocular pressure

## Abstract

Glaucoma is conventionally defined as a chronic optic neuropathy characterized by progressive loss of retinal ganglion cells (RGCs) and optic nerve fibers. Although glaucoma is often associated with elevated intraocular pressure (IOP), significant IOP reduction does not prevent progression of the disease in some glaucoma patients. Thus, exploring IOP-independent mechanisms of RGC loss is important. We describe chronic systemic administration of aldosterone and evaluate its effect on RGCs in rat. Aldosterone was administered via an osmotic minipump that was implanted subcutaneously into the mid-scapular region. Although systemic administration of aldosterone caused RGC loss associated with thinning of the retinal nerve fiber layer without elevated IOP, the other cell layers appeared to be unaffected. After chronic administration of aldosterone, RGC loss was observed at 2 weeks in the peripheral retina and at 4 weeks in the central retina. However, administration of mineralocorticoid receptor blocker prevented RGC loss. These results demonstrate aldosterone is a critical mediator of RGC loss that is independent of IOP. We believe this rat normal-tension glaucoma (NTG) animal model not only offers a powerful system for investigating the mechanism of neurodegeneration in NTG, but can also be used to develop therapies directed at IOP-independent mechanisms of RGC loss.

Although therapeutic methods aimed at alleviating the basic pathologic process of normal-tension glaucoma (NTG) have yet to be established, there seems to be little doubt that intraocular pressure (IOP) is a risk factor in most patients.^1, 2, 3, 4^ However, a reduction in IOP does not prevent progression in patients with NTG,^5^ which indicates that factors other than an elevated IOP are involved in the progression of glaucoma.^6^ The association of glaucoma with various systemic vascular diseases including low systemic blood pressure, transient nocturnal decreases in blood pressure, hypertension, migraine, vasospasm, and diabetes has been reported.^6, 7, 8, 9^ Many patients with chronic open-angle glaucoma have coexisting vascular disorders, with the most common finding reported to be systemic hypertension, which occurs in 48% of the total chronic open-angle glaucoma population.^10^ All of these observations suggest the possibility that factors not dependent on the IOP may contribute to the disease progress. Therefore, elucidation of such factors is necessary if we are to understand the pathogenesis of glaucoma and in order to be able to further guide efforts toward improved therapeutics.

The renin–angiotensin–aldosterone system (RAAS) is a major controller of systemic blood pressure. Abnormal activation of this system has been postulated to participate in the occurrence of end-organ damage in hypertensive patients.^11, 12^ Aldosterone is a steroid hormone that elicits its effects by binding to the mineralocorticoid receptor (MR). Although many studies have investigated the role of angiotensin II (Ang II) in mediating cardiovascular damage, relatively little attention has been paid to the role of aldosterone, which is the end product of the RAAS. However, there are data that suggest aldosterone may have an important role in the pathogenesis of cardiovascular diseases, a role that is independent of Ang II. As compared with patients with essential hypertension, patients with primary aldosteronism, in which Ang II levels are usually very low, have a higher incidence of left ventricular hypertrophy,^13^ albuminuria,^14^ and stroke.^15, 16^ Furthermore, experimental animal data support a role for aldosterone in mediating cardiovascular injury in the kidney and brain.^17, 18^

We recently reported that the expression of angiotensin II type 1 receptor (AT1-R) in the retina is increased after ischemia-reperfusion^19, 20^ and that treatment with an angiotensin-converting enzyme (ACE) inhibitor, AT1-R blocker (ARB), and MR antagonist reduced retinal ischemia-reperfusion injury.^19, 21^ Moreover, we have also shown that intravitreal injection of aldosterone reduced the number of retinal ganglion cells (RGCs).^20^ To investigate the role of aldosterone in mediating retinal injury, we infused aldosterone in rats using an experimental model in which an osmotic minipump was subcutaneously implanted at the dorsum of the neck.

## Results

### Loss of RGCs in aldosterone-treated rats

**Figure 1:**
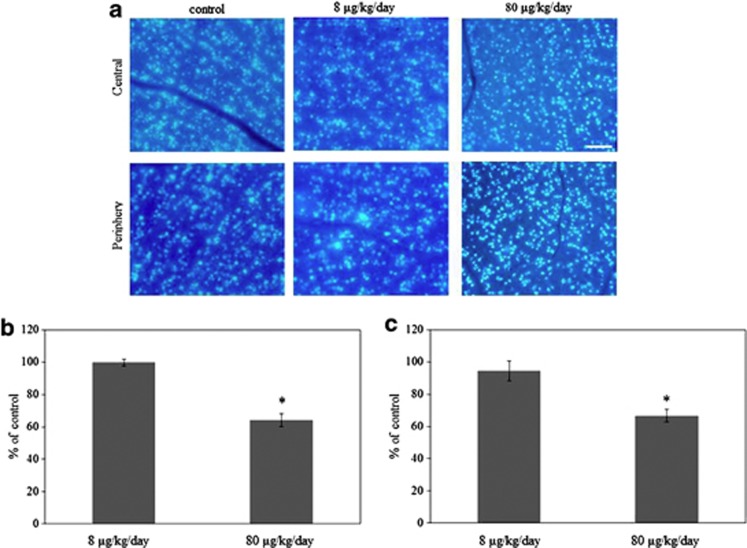
Effect of aldosterone on retinal ganglion cell (RGC) death. (**a**) Retrograde labeling of RGCs treated with 8 *μ*g/kg/day or 80 *μ*g/kg/day aldosterone for 6 weeks, and a normal control eye. Micrographs of the central and peripheral areas were taken ∼1 and 4 mm away from the optic nerve head. Scale bar, 100 *μ*m. RGCs were counted in the central (**b**) and peripheral (**c**) areas. Results are expressed as the mean±SEM (*n*=4 in each group). *P<0.001 *versus* control (Dunnett's multiple comparison test)

Figure 1a shows representative results for the RGC labeling in the 8 *μ*g/kg/day, 80 *μ*g/kg/day aldosterone- and vehicle-treated rats for 6 weeks. The number of RGCs were similar between the vehicle- and 8 *μ*g/kg/day aldosterone-treated rats in the central (99.6±2.0%, *P*=0.99) and peripheral retina (94.3±6.2%, *P*=0.56). However, RGCs were decreased in the 80 *μ*g/kg/day aldosterone-treated rats in the central (64.2±3.9%, *P*<0.001) and peripheral retina (66.7±3.9%, *P*=0.001, *n*=4).

**Figure 2:**
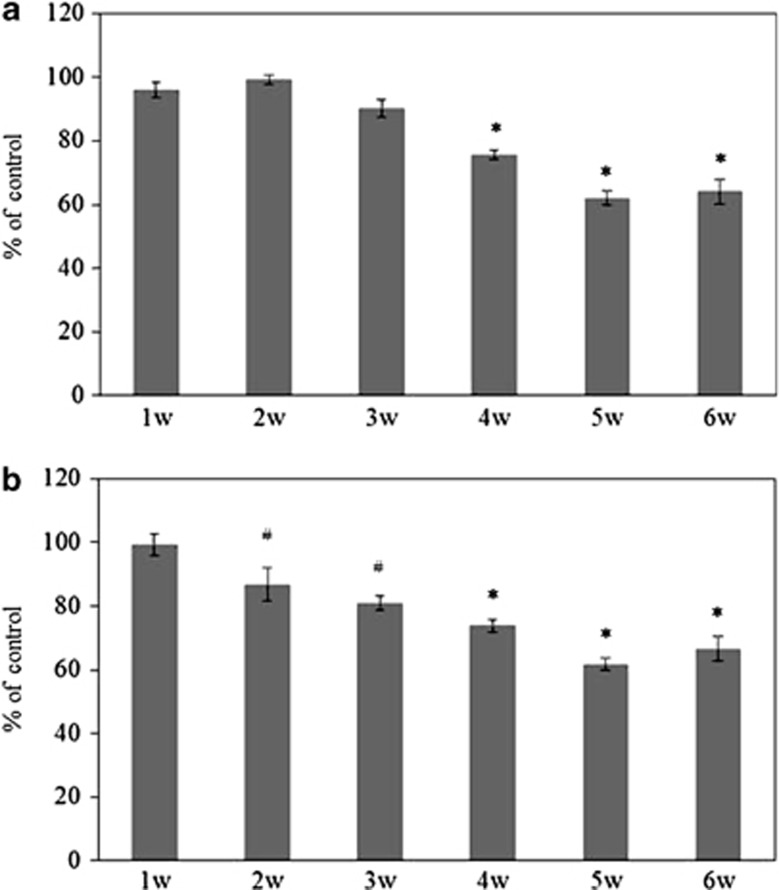
The number of RGCs in the eyes treated weekly with 80 *μ*g/kg/day aldosterone. (**a**) In the central retina, RGC was decreased 4 weeks after the administration of aldosterone. (**b**) In the peripheral retina, however, reduction in the number of RGCs was already detected at 2 weeks after the administration of aldosterone. Results are expressed as the mean±S.E.M. (*n*=4 in each week). ^#^*P*<0.05, *P<0.001 *versus* control (Dunnett's multiple comparison test)

To determine the time point where the RGCs decreased in aldosterone-treated rats, the numbers of RGCs were counted every week. At 2 weeks after the continual administration of aldosterone, RGCs were significantly decreased in the peripheral retina (*P*=0.03), whereas the cell density for the central RGCs showed no significant decrease (*P*>0.99) (Figure 2). At 4 weeks after continual administration of aldosterone, RGCs were decreased not only in the peripheral but also in the central retina (*P*<0.001, *n*=4 for each week). These data suggested that in aldosterone-treated rats, the tendency for neuronal degeneration in the ganglion cell layer initially started from the peripheral retina and then spread to the central retina.

**Figure 3:**
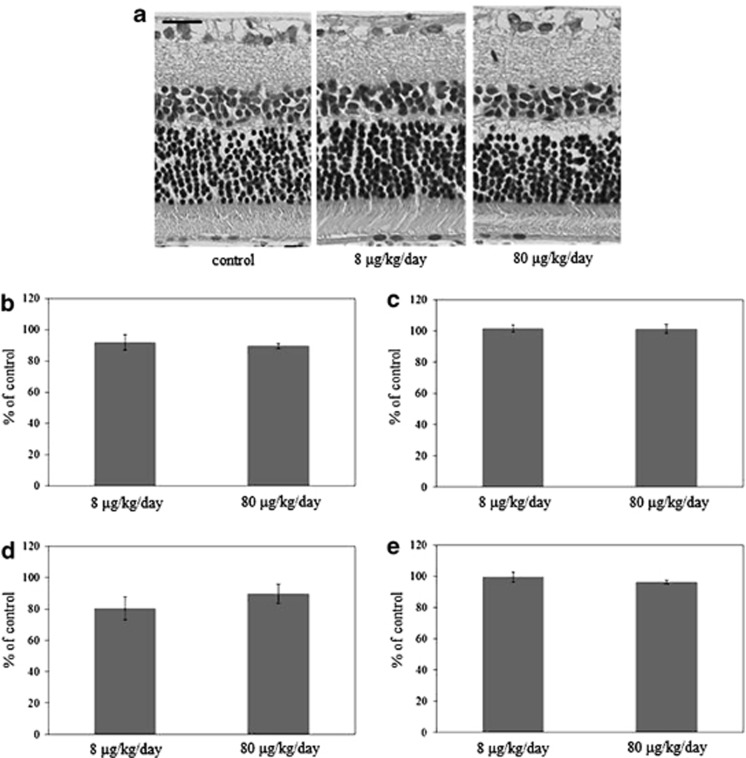
Retinal thickness layer analysis. (**a**) Light micrographs of the retina of an eye treated with 8 *μ*g/kg/day or 80 *μ*g/kg/day aldosterone for 6 weeks, and a normal control eye. Change in mean thickness of the (**b**) inner plexiform layer (IPL), (**c**) inner nuclear layer (INL), (**d**) outer plexiform layer (OPL), and (**e**) outer nuclear layer (ONL). Results are expressed as the mean±S.E.M. (*n*=4 in each group). Scale bar, 20 *μ*m

To determine if there was degeneration of any of the other retinal neurons, we performed a retinal layer thickness analysis. Thickness measurements in animals treated with 8 *μ*g/kg/day aldosterone for 6 weeks were 92.0±5.0% (*P*=0.64) for the inner plexiform layer (IPL), 101.6±2.3% (*P*=0.87) for the inner nuclear layer (INL), 80.2±7.3% (*P*=0.46) for the outer plexiform layer (OPL), and 99.4±3.2% (*P*=0.98) for the outer nuclear layer (ONL) (Figure 3). In animals treated with 80 *μ*g/kg/day aldosterone for 6 weeks, the thicknesses were 89.5±1.7% (*P*=0.47) for IPL, 101.3±2.9% (*P*=0.90) for INL, 89.6±6.2% (*P*=0.79) for OPL, and 96.3±1.0% (*P*=0.64) for ONL (*n*=4 in each group). These findings suggested that aldosterone-treated rats showed a severe neuronal loss only in the ganglion cell layer.

### Degeneration of optic nerve in aldosterone-treated rats

**Figure 4:**
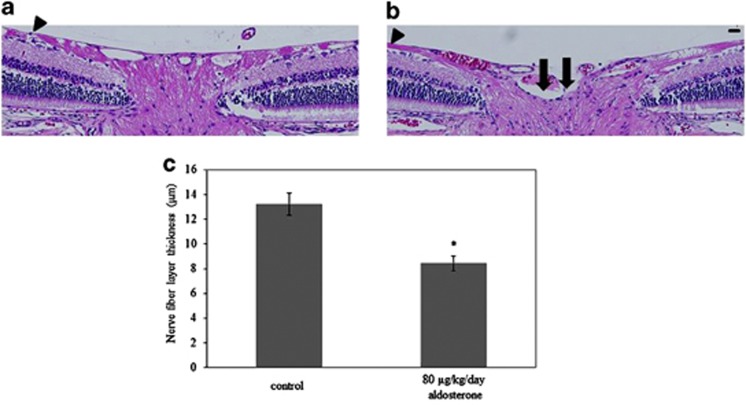
Optic nerve degeneration in aldosterone-treated rats. Representative photos of HE-stained sections in normal (**a**) and aldosterone-treated (**b**) rats. Sections illustrate the thinning of the retinal nerve fiber layer (arrowheads) in the aldosterone-treated rats as compared with normal rats. Cupping extends to the posterior aspect of the inner retinal layer (allows). Scale bar, 20 *μ*m. (**c**) Quantitative analysis was performed in order to show the significant difference in the thickness of the nerve fiber layer in the aldosterone-treated rats. Results are expressed as the mean±S.E.M. (*n*=4 in each group). *P<0.01 *versus* control (independent Student's *t*-test)

Degeneration of the optic nerve is one of the hallmarks of glaucoma. In association with the progressive loss of RGCs, rats treated with aldosterone for 6 weeks showed attenuation of the retinal nerve fiber layer (Figure 4b) compared with controls (Figure 4a). Quantitative analysis showed there was significant nerve fiber layer thinning in the aldosterone-treated rats (13.2±0.9 *μ*m, *P*=0.004) at the central retina (200 *μ*m from the optic nerve head) when compared with normal rats (Figure 4c) (8.4±0.6 *μ*m, *n*=4 in each group). Consistent with severe RGC loss, optic nerve cupping was also apparent in the aldosterone-treated rats.

### Normal IOP in aldosterone-treated rats

**Figure 5:**
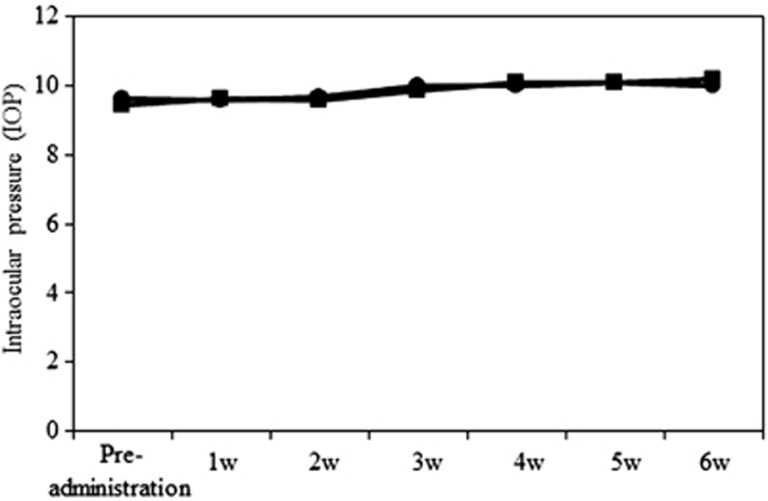
Normal intraocular pressure (IOP) in aldosterone-treated rats. There were no significant differences in the IOP between the vehicle- and 80 *μ*g/kg/day aldosterone- treated rats. ●: vehicle-treated rats, ▪: aldosterone-treated rats. Results are expressed as the mean±S.E.M. (*n*=4 in each week)

To determine whether the IOP was affected in aldosterone-treated rats, IOP was measured every week. No significant differences in the IOP levels were detected between the vehicle- and aldosterone-treated rats (Figure 5) (*P*=0.09∼0.99, *n*=4 in each week). These results suggested that RGC loss in aldosterone-treated rats was IOP-independent.

### Effect of spironolactone on RGC survival

**Figure 6:**
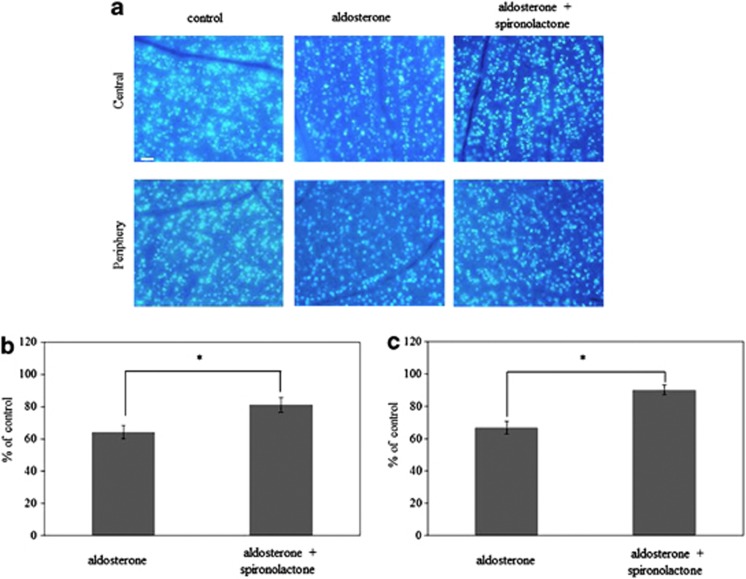
Effect of spironolactone on aldosterone-induced retinal ganglion cell death. (**a**) Retrograde labeling of RGCs in a normal control eye and aldosterone-treated rats with or without spironolactone for 6 weeks. Scale bar, 100 *μ*m. RGCs were counted in the central (**b**) and peripheral (**c**) areas. Results are expressed as the mean±S.E.M. (*n*=4 in each group). *P<0.05 *versus* aldosterone-treated rats (Tukey's honestly significant difference test)

To assess aldosterone involvement in RGC loss, we next examined the effect of the MR antagonist spironolactone on RGC loss in the aldosterone-treated rat. Figure 6a shows representative results of the RGC labeling in rats treated with aldosterone alone and those treated with the combination of spironolactone with aldosterone for 6 weeks. As shown in Figures 6b and c, administration of spironolactone significantly prevented RGC loss in the central (81.1±4.4%, *P*=0.02) and the peripheral retina (90.0±2.9%, *P*=0.001) (*n*=4 in each group). The protective effect of the MR antagonist suggests that aldosterone is partly involved in RGC loss.

## Discussion

Systemic administration of aldosterone resulted in progressive RGC loss and glaucomatous optic nerve degeneration without elevated IOP. The results of this study raise the possibility that aldosterone may very well have a role in RGC death in human NTG.

MR is expressed in RGCs and in the cells of the inner nuclear layer in the normal retina.^22, 23^ Many studies have previously demonstrated that Ang II, the principal effector of the RAAS, induces cellular changes through NADPH oxidase-mediated reactive oxygen species (ROS) production.^24, 25, 26^ We have previously reported that the expression of AT1-R increases 12 h after reperfusion.^19, 21^ ROS production after 12-h reperfusion is mediated via a NADPH oxidase pathway.^21^ However, the combined treatment of aldosterone with the AT1-R antagonist, candesartan, provided no protective effect against the retinal ischemia-reperfusion injury.^20^ Thus, it may be the aldosterone that has a critical role in this ischemia-reperfusion model. Moreover, these results suggest that retinal ischemic injury might occur due to ROS production via the local RAAS. It has been reported that aldosterone induces apoptosis of proximal tubular cells,^27^ mesangial cells^28^ and cardiac myocytes^29^ in a ROS-dependent manner. On the basis of these findings, we assume that RGC death in our model may be induced by aldosterone in a ROS-dependent manner.

Ischemia/inflammation may have a role in the pathogenesis of NTG because of an increase in the plasma endothelin-1 and the macrophage chemoattractant protein 1 (MCP-1).^30^ Aldosterone is a potent stimulator of inflammation.^31, 32^ Wilkinson-Berka *et al.*^22^ recently reported that retinal MCP-1 mRNA and protein were modulated by aldosterone, which was reduced by spironolactone. On the basis of these findings, they speculated there was a pathogenic role for MR-aldosterone in retinal inflammation.

Although intravitreal injection of aldosterone reduced the number of RGCs, it did not affect other retinal neurons.^20^ A previous study has reported finding elevated plasma aldosterone levels when 0.66 *μ*g/h aldosterone was administered subcutaneously by implanted osmotic pumps.^18^ Moreover, systemic administration of 0.75 *μ*g/h aldosterone was found to exacerbate the pathological neovascularization in experimental retinopathy of prematurity.^22^ In our study, systemic administration of 80 *μ*g/kg/day, that is, 0.67–0.83 *μ*g/h, aldosterone significantly reduced the number of RGCs. Although we did not investigate the blood-retinal-barrier penetration of aldosterone, we assume that the effective concentration of aldosterone did reach the retina in our study.

Primary aldosteronism in the essential hypertensive population has been reported to range from 5 to 15%, with the overall incidence most likely to be around 10%.^33, 34^ Although hypertension has been associated with glaucoma in some population-based studies,^35, 36, 37^ other prospective studies have failed to verify any association between incident glaucoma and the systolic or diastolic blood pressures.^38, 39^ This could be attributed to the distribution of age or racial variability, with a higher susceptibility to open-angle glaucoma among hypertensive white as compared with black subjects. Another possible explanation for this discrepancy is that one study could have included fewer patients with hypertension due to primary aldosteronism in relation to the other study.

In conclusion, the RGC loss observed after systemic administration of aldosterone was a time-dependent loss that occurred without any elevation in the IOP. We believe this animal model offers a powerful system for investigating mechanisms of neurodegeneration in NTG and for developing therapies directed at the IOP-independent mechanism of RGC loss. Further clinical studies will need to be undertaken to clarify the specific relationship between NTG and primary aldosteronism.

## Materials and Methods

### Animals

Male Sprague–Dawley rats weighing 200 to 250 g were obtained from Charles River Japan (Yokohama, Japan). Rats were permitted free access to standard rat food (Oriental Yeast Co., Ltd., Tokyo, Japan) and tap water. Animal care and all experiments were conducted in accordance with the approved standard guidelines for animal experimentation of the Kagawa University Faculty of Medicine and adhered to the ARVO Statement for the Use of Animals in Ophthalmic and Vision Research.

### Drugs

Aldosterone and spironolactone were obtained from Sigma-Aldrich (St. Louis, MO, USA). Aldosterone was dissolved in dimethyl sulfoxide (DMSO) to produce the stock solutions, which were then diluted to the final required concentrations. The final DMSO concentration never exceeded 5%. After dissolving 10 mg/kg/day spironolactone^20^ in carboxymethyl cellulose (CMC) to produce the stock solution, feeding needles were then used to orally administer the solution to each of the animals. The final CMC concentration never exceeded 0.5%. For the control group, additional animals were treated with vehicle only (0.5% CMC in PBS).

### Experimental animals

Aldosterone (8 or 80 *μ*g/kg/day) or vehicle was administered to the experimental animals using a subcutaneous osmotic minipump (Alzet model 2006, DURECT Corporation, Cupertino, CA, USA). The minipumps were implanted subcutaneously into the mid-scapular region.

### Histological examination

For the histological examination, rats were anesthetized by intraperitoneal injection of pentobarbital sodium (50 mg/kg) at 6 weeks after the systemic administration of aldosterone and then perfused intracardially with phosphate-buffered saline (PBS), followed by perfusion with 4% paraformaldehyde in PBS. Subsequently, the anterior segments, including the lens, were removed. The posterior eyecups were then embedded in paraffin, and thin sections (5-*μ*m thickness) were cut using a microtome. Each of the sections was carefully cut to include the full length from the superior to inferior along the vertical meridian through the optic nerve head. Each eye was then mounted on a silane-coated glass slide and stained with hematoxylin and eosin (HE). Scleral thickness was measured to confirm that the sections were not oblique sections.

Five sections were randomly selected in each eye. One investigator with no prior knowledge of the treatments administered was responsible for performing all of the light microscopic examinations (magnification; 10 × 100; Olympus BX-51, Olympus Inc., Tokyo, Japan). A microscopic image of each section within 0.5 to 1 mm superior of the optic disc was scanned. In each computer image, the thickness of the IPL, INL, OPL, and ONL were measured.

HE-stained retinal sections with the optic nerve stump were used to examine the morphology of the optic nerve head.

### Retrograde labeling of retinal ganglion cells

At 7 days before sacrifice, Fast Blue (Polysciences Inc., Warrington, PA, USA) was injected bilaterally into the superior colliculi of anesthetized rats. The skull was exposed and kept dry and clean. After identifying and marking the bregma, a small window was drilled in the scalp in both the right and left hemispheres. The windows were drilled to a depth of 3.6 mm from the surface of the skull and located at 6.8 mm behind the bregma on the anteroposterior axis, and 1.5 mm lateral to the midline. Using a Hamilton syringe (Hamilton Co., Reno, NV, USA), 1.5 *μ*l of 3% Fast Blue was slowly injected into the bilateral superior colliculi. After suturing the skin over the wound, antibiotic ointment was applied.

### Tissue preparation and assessment of RGC survival

Animals were sacrificed using an overdose of pentobarbital at 1 week after fluorescent dye application. Whole, flat-mounted retinas were then assayed for RGC density. Rat eyes were enucleated and fixed in 4% paraformaldehyde for 10 h at room temperature. After removal of the anterior segments, the resultant posterior eyecups were left in place. Subsequently, four radial cuts were made in the periphery of each eyecup, with the retina then carefully separated from the retinal pigment epithelium. To prepare the flat mounts, the retina was dissociated from the underlying structures, flattened by making four radial cuts, and then spread on a gelatin-coated glass slide. Labeled RGCs were visualized under a fluorescence microscope (Olympus BX-51/DP-72, Olympus, Tokyo, Japan) with an ultraviolet filter (excitation filter, 330–385 nm; barrier filter, 420 nm). Fluorescence-labeled RGCs were counted in 12 microscopic fields of retinal tissue from two regions in each quadrant at two different eccentricities, 1 mm (central) and 4 mm (peripheral) away from the optic disc. Image-Pro Plus software (Version 4.0, Media Cybernetics, Bethesda, MD, USA) was used to count the total number of RGCs in each eye.

### IOP measurement

Rats were anesthetized using an intraperitoneal injection of 50 mg/kg pentobarbital sodium prepared at room temperature. Subsequently, topical 0.4% oxybuprocaine (Benoxyl; Santen Pharmaceuticals, Osaka, Japan) was then applied to both eyes.

An osmotic minipump was subcutaneously implanted at the dorsum of the neck and used to infuse the aldosterone. The IOP was measured before and every week after the implantation. All of the IOP measurements were made by a TonoLab tonometer (TioLat, Inc., Helsinki, Finland), which recorded the mean of six readings using the optimal variability grade.

### Statistical analysis

All data are presented as the mean±S.E.M.. Data were analyzed using an independent Student's *t-*test, Dunnett's multiple comparison test, or Tukey's honestly significant difference test, as appropriate. Statistical analyses were performed using SPSS version 19.0 (SPSS Inc., Chicago, IL, USA). A *P*-value of <0.05 was considered statistically significant.

## Abbreviations

NTG: normal-tension glaucoma
IOP: intraocular pressure
RAAS: renin–angiotensin–aldosterone system
MR: mineralocorticoid receptor
Ang: angiotensin
AT1-R: angiotensin II type 1 receptor
ACE: angiotensin-converting enzyme
ARB: angiotensin II type 1 receptor blocker
RGC: retinal ganglion cell
IPL: inner plexiform layer
INL: inner nuclear layer
OPL: outer plexiform layer
ONL: outer nuclear layer
ROS: reactive oxygen species
MCP-1: macrophage chemoattractant protein 1
DMSO: dissolved in dimethyl sulfoxide
CMC: carboxymethyl cellulose
HE: hematoxylin and eosin
